# Nature as a Commodity: What's Good for Human Health Might Not Be Good for Ecosystem Health

**DOI:** 10.3389/fpsyg.2018.01673

**Published:** 2018-09-10

**Authors:** Yolanda van Heezik, Eric Brymer

**Affiliations:** ^1^Zoology Department, University of Otago, Dunedin, New Zealand; ^2^Institute of Sport, Physical Activity and Leisure, Leeds Beckett University, Leeds, United Kingdom

**Keywords:** nature-related wellbeing, urban biodiversity, nature therapy, nature doses, greenspaces, nature exposure

Are you getting enough Vitamin N? Richard Louv (2008) coined this term in his book “Last Child in the Woods,” in response to growing evidence that suggests humans are increasingly disinterested with, and disconnected from the natural world. Concurrent with the literature on the extent of disconnection (Miller, 2005; Soga et al., 2016; van Heezik and Hight, 2017) is an ever-expanding body of literature documenting the many psychological, physical, and spiritual health benefits derived from nature contact (Keniger et al., 2013; Bratman et al., 2015; Martyn and Brymer, 2016; Frumkin et al., 2017). In fact human survival is inextricably linked with nature: the species and their inter-relationships that make up the fabric of ecosystems function to sustain all life on Earth. Biodiversity in all landscapes, including urban ones, provides humans with essential ecosystem services, such as food provisioning, climate and flood regulation, nutrient cycling, carbon sequestration, and pollution reduction (Elmqvist et al., 2015). Frameworks have been proposed for evaluating the economic value of biodiversity (Edwards and Abivardi, 1998), with more recent approaches acknowledging the inter-play between social well-being, economic sustainability, and biodiversity and ecosystem function (Tzoulas et al., 2007; Laurila-Pant et al., 2015). These socio-cultural valuation techniques recognize that biodiversity provides society with benefits, such as mental well-being, ethical, spiritual and cultural values, as well as economic values. Psychological well-being benefits have been positively associated with the number of species perceived by people in the environments around them (Fuller et al., 2007; Dallimer et al., 2012). Loss of biodiversity reduces the efficiency by which ecological communities perform ecosystem services, as well as the stability of ecosystem function over time (Cardinale et al., 2012).

The role that urban nature can play in enhancing psychological and physical well-being and reducing health-related costs could be seen by those advocating for the protection and restoration of urban biodiversity and ecosystem function as a positive outcome, suggesting a need to place greater value on biodiverse urban spaces. Another, less positive, scenario is that the connection between human health and nature might threaten the ecological integrity of urban green spaces by commodifying nature, especially if green spaces are designed and managed for human health benefits alone, with little concern for supporting biodiversity or ecosystem services. In this latter scenario nature could become a “pill” with only those aspects of nature that most strongly influence human health and wellbeing considered to be important in the design process. Here we demonstrate how this undesirable outcome might be realized, and argue that a focus on treating urban nature purely as an efficient means of delivering minimal levels of psychological well-being is short-sighted. The development of knowledge and implementation of best practice that ensures outcomes that provide for psychological well-being requires an interdisciplinary approach that encourages diverse ecological communities with greater input by ecologists.

## Nature as a therapeutic device

Mounting support for the link between contact with nature and improved human health and well-being has led to nature being applied for therapeutic purposes; for example, Shinrin-yoku or “forest bathing” in Japan (Song et al., 2016; Hansen et al., 2017), horticultural therapy or gardening (e.g., Clatworthy et al., 2013; Kamioka et al., 2014), participation in woodland management (Townsend, 2006), and green prescriptions (Van den Berg, 2017). A systematic review of studies of nature-assisted therapy revealed robust support for its effectiveness (Annerstedt and Währborg, 2011). Evidence suggests that time in nature is particularly beneficial for psychological health (Brymer et al., 2014; Bragg and Atkins, 2016). Consequently, some researchers have been focusing on identifying the minimum “doses” of nature needed to benefit well-being (Shanahan et al., 2015; Cox et al., 2017).

## Minimum doses for well-being benefits; what might they mean for biodiversity?

How much nature exposure is required to derive a psychological health benefit? Shanahan et al. (2016) focused on time spent by people in nature and applied a dose-response analysis, used in health contexts to evidence effectiveness: they found that visits of 30+ min to green spaces could reduce the population prevalence of depression and high blood pressure by 7 and 9%, respectively, translating to savings for public health budgets. Such evidence has influenced health-related decision-making globally. In general, dose-response calculations have influenced physical activity research and manifest as green prescriptions by doctors, whereby people are encouraged to be more active, and green spaces are promoted as beneficial. From this perspective, psychological health benefits come about directly from the fact that green spaces encourage physical activity. However research also indicates green spaces have direct positive effects on psychological health and well-being (Pretty et al., 2006; Barton et al., 2016). Green prescriptions can therefore be an important contribution to public health, and strategies to encourage adoption of green prescriptions have been proposed (Van den Berg, 2017).

While green prescriptions and recommendations on the frequency and duration of exposure to nature might seem helpful, or at least benign, Stanley et al. (2015) argue that considering nature in this way has detrimental consequences for biodiversity. Specifically, the growing numbers of people accessing green spaces only for health benefits, together with the promotion of health-related (including exercise) requirements within green space design, threatens biodiversity and the integrity of urban ecosystems. This is because green spaces are inevitably modified to accommodate human use. Examples include, pathways extended and widened, large flat areas (e.g., lawns) created for exercise groups, vegetation modified to enhance users' perceptions of safety, and artificial lighting installed for use outside daylight hours (Stanley et al., 2015). Habitat design, if undertaken purely from a health and well-being perspective, might exclude species perceived as undesirable, such as snakes or spiders. Often these green spaces are rated on aesthetic characteristics and because aesthetic preferences do not always align with habitat supporting biodiversity, recreational spaces might provide resources for only the most tolerant urban exploiters, which are often non-native (McKinney, 2002). Less tolerant species are likely to abandon popular, well-lit areas when frequent noise interferes with auditory cues, when sounds are perceived as threats, and when pedestrians and dogs interrupt foraging, resulting in more time being vigilant, energy wasted, and foraging opportunities lost. While urban green spaces might provide habitat for some hardy non-human residents, paradoxically “people-friendly” spaces are not necessarily “wildlife-friendly.” Thus design of green spaces might need to consider a broader perspective than aesthetic characteristics or the maximization of recreation activities.

Others have applied dose-response curves to estimate the minimum levels of vegetation required for improved well-being. Cox et al. (2017) evaluated five neighborhood nature characteristics and calculated dose-responses for mental disorders, concluding that quantifiable reductions in the prevalence of poor mental health could be achieved with even low levels of components of neighborhood nature. Another study investigated the dose of nature required to reduce stress in people subjected to a Trier Social Stress Test. Study participants watched assigned street scenes with different tree densities; the male dose-response curve indicated that stress reduction was greatest at tree densities of 24–34% (Jiang et al., 2014).

While these studies provide valuable insights into the amount and type of nature exposure necessary to effect improved human well-being, this approach becomes problematic when the minimal levels and type of vegetation identified as safe and adequate to enhance human well-being are insufficient to support biodiverse communities and stable ecosystem function. Tall trees and shrub understoreys provide habitat for small mammals (Dickman and Doncaster, 1987), birds (Jokimäki and Suhonen, 1993; van Heezik et al., 2008), and invertebrates (Smith et al., 2006), and are an important generator of ecosystem services (Gaston et al., 2013). Despite the important role that vegetation volume plays in supporting biodiversity and ecosystem services, trade-offs, and conflicts exist between planning for biodiversity and planning for local residents. For example, these same rich biodiverse habitats might also present health and safety issues (e.g., dark parks, health problems from pollen, places for drug taking activities). It is therefore feasible that those responsible for greening urban environments might introduce vegetation based on an easy-to-manage approach, rather than an approach that considers local biodiversity and ecosystem services.

## What kind of nature?

Keniger et al. (2013) emphasized the importance of understanding the characteristics of natural settings that trigger well-being benefits and how these vary among cultural and socioeconomic groups. However the kind of nature researchers have focused on to demonstrate links to positive well-being responses is frequently not reflective of the type of natural environments conservationists seek to encourage. In many studies on psychological well-being benefits the natural environment is described as parkland with scattered shrubs and trees (Bowler et al., 2010; Bratman et al., 2015). Descriptions of “nature treatments” can be very broad. From a health perspective the notions of greenness and nature often stem from what the environment looks like, and biodiversity is either assumed because the environment looks green or not considered at all (Keniger et al., 2013; Shwartz et al., 2014; Sandifer et al., 2015). This is because few studies specifically focusing on health have involved ecologists. “Greenness” is measured remotely if the focus of the study is on entire neighborhoods (e.g., Beyer et al., 2014; Dadvand et al., 2016) or using generalized land-use databases (e.g., Alcock et al., 2014; Gidlow et al., 2016). These are convenient to use in population-level studies, but do not represent many of the relevant features of the greenness, such as species diversity and composition, vertical structure, and wildness. In a review of 125 journal articles about green space, fewer than half defined what the green spaces consisted of with only simple generic descriptions provided; e.g., park, golf course (Taylor and Hochuli, 2017). Moreover, when greenspace quality was referred to, “quality” was subjectively determined without reference to ecological integrity.

## Does using nature to enhance human well-being compromise biodiversity?

Urban nature is increasingly seen as a manageable resource to enhance human well-being. By viewing nature as a commodity that supplies health benefits, and by identifying minimum amounts needed to gain benefits, we risk trivializing a deep affective response to nature. We might end up with a watered-down, biodiversity-poor version of nature with compromised ecosystem services. By creating a new baseline of what is considered normal we could exacerbate ongoing shifts toward more depleted environments. The concept of shifting baselines (Pauly, 1995) is pertinent to each generation of urban residents that perceive the state of the environments they encounter in their childhood as normal, unaware of the past losses and the depleted and altered nature of the biodiversity that remains. Paradoxically, this could also reduce the psychological benefits from human-nature interactions.

Research linking psychological wellbeing and nature has traditionally focused on the individual psychological workings of the individual or the form and structure of nature (e.g., color, objects and spaces between objects), often arguing for “greenness” as the mediator for wellbeing (Brymer et al., 2014). If well-being benefits, albeit minimal, can be gained from highly modified, simplistic greenspaces, and these types of green spaces become the new norm for the next generation, then there will be little incentive to restore greenspaces to a more natural biodiverse state, or even to protect what we currently have from degradation.

While it is still early days, in recent years research into human health is acknowledging that the focus on “greenness” is too simplistic and, when considering psychological well-being, the “richness” of the environment and the human-nature relationship is turning out to be of paramount importance (Brymer et al., 2014; Fabjanski and Brymer, 2017; Lawton et al., 2017). Therefore, an interdisciplinary approach, including input from ecologists and health professionals, is essential to optimize green space design for psychological well-being, which will also ensure ecosystem well-being. A consensus on greenspace definitions is necessary to provide a context for such research (Taylor and Hochuli, 2017). Tools such as the “Bioscore” developed by Hand et al. (2016), which incorporates perceived diversity and human perceptions of naturalness, might be applied to a variety of greenspaces (e.g., Müller et al., 2018). Viewing nature as a “pill,” separate from humanity but applied as required, is short-sighted. More meaningful gains for human well-being can be achieved through recognition that the artificial divide between people and nature is false. Developing a culture of stewardship rather than one of exploitation, and lifting biodiversity baselines through ecological restoration is necessary. From a psychological health perspective, what is urgently needed is a principled theoretical framework, combining ecological, and psychological related knowledge that can guide a more enlightened program of research and practice. Only through this interdisciplinary approach, and the development of frameworks that support this approach, will we promote and protect the health and well-being of people and of nature.

## Author contributions

YvH conceptualized the opinion piece and wrote a first draft. EB contributed to concept and refined the manuscript.

### Conflict of interest statement

The authors declare that the research was conducted in the absence of any commercial or financial relationships that could be construed as a potential conflict of interest.
